# Reporting of primary endpoint and associated spin in randomized myeloma trials

**DOI:** 10.1093/oncolo/oyag221

**Published:** 2026-06-02

**Authors:** Mitch Singstock, Abul Hasan Shadali Abdul Khader, Cole Wayant, Mackenzie Lemieux, Maria Mainou, Ghulam Rehman Mohyuddin

**Affiliations:** Department of Internal Medicine, University of Utah, Salt Lake City, UT, 84112, United States; Department of Internal Medicine, Mercy Catholic Medical Center, PA, 19023, United States; Division of Medical Oncology, University of Colorado Anschutz, Denver, CO, 80045, United States; Department of Internal Medicine, University of Utah, Salt Lake City, UT, 84112, United States; Clinical Research and Evidence Based Medicine Unit, Aristotle University of Thessaloniki, Thessaloniki, 54124, Greece; Division of Hematology, Huntsman Cancer Institute, University of Utah, Salt Lake City, UT, 84112, United States

**Keywords:** multiple myeloma, randomized controlled trials, spin, primary endpoint, research reporting, sample size calculation, trial interpretation, research integrity

## Abstract

**Background:**

Oncology clinical trials, regardless of success in meeting their primary endpoint, often suffer from spin—the misrepresentation of research findings. Despite dramatic improvements in myeloma outcomes, numerous recent myeloma trials have failed to meet their primary endpoints while others have amplified marginally positive findings. A thorough evaluation of reporting practices and spin in myeloma randomized controlled trials (RCTs) is needed.

**Materials and Methods:**

We performed a cross-sectional analysis of myeloma RCTs that began enrollment in January 2015 or later, with final search conducted in March 2025. Reporting of prespecified primary endpoints and sample size calculations was abstracted. Two reviewers screened for the presence of spin, defined as at least one reporting practice that could mislead readers by emphasizing benefit or minimizing unfavorable findings. Multivariable logistic regression was used to determine factors associated with spin. The methods were registered on the Open Science Framework.

**Results:**

Of 82 screened studies, 71 RCTs were included. The prespecified primary endpoint was statistically significant in 46 trials (64.8%). Sample size calculations were reported in 51 trials (71.8%), and conclusions aligned with primary endpoint results in 63 trials (88.7%). Spin occurred in 25 trials (35.2%), most commonly through optimistic language and emphasis on secondary/subgroup findings. In multivariable analysis, meeting the prespecified primary endpoint was associated with lower odds of spin (adjusted odds ratio [OR] = 0.15; 95% CI, 0.03-0.70; *P* = .016).

**Conclusion:**

Spin affected one-third of contemporary myeloma RCTs and was strongly associated with nonsignificant primary endpoints, highlighting the need for primary endpoint-aligned interpretation and transparent sample size reporting.

Implications for Practice In myeloma randomized controlled trials, interpretive spin is common when primary endpoints are not met. Authors, reviewers, and editors should require that abstracts and conclusions explicitly report prespecified primary endpoint results, avoid optimistic framing of nonsignificant findings, and clearly label secondary/subgroup analyses as exploratory with multiplicity caveats. Improved reporting of sample size assumptions can help readers distinguish negative from underpowered trials.

## Introduction

Randomized controlled trials (RCTs) are fundamental to therapeutic advancement in oncology, with prespecified primary endpoints serving as the anchor for trial design, statistical interpretation, and comparison across studies.^1^ In myeloma, therapeutic advances have been accompanied by an increasingly complex trial landscape, with frequent reliance on multiple surrogate endpoints, increasing the likelihood of distortion of results by focusing on outcomes other than the primary endpoint.^2^^,^^3^ Misrepresentation of research findings, defined as spin, is widespread in oncology studies, and has not been previously systematically studied in myeloma.^4^

Among cancer RCTs with nonsignificant primary endpoints, approximately 40%-60% of abstracts and/or conclusions contain spin, most commonly through selective emphasis on secondary or subgroup findings.^4^^,^^5^ Spin has been observed in both industry-sponsored and investigator-initiated studies and may be further amplified through press releases and news coverage.^6–8^ Prior methodological work has linked spin to overestimation of benefit, underappreciation of toxicity, biased interpretation of evidence, and research waste, with downstream effects on guideline language, reimbursement deliberations, and trial prioritization.^9^^,^^10^

In myeloma, trials frequently assess correlated efficacy measures such as progression-free survival (PFS), response depth, or measurable residual disease, creating opportunities for favorable findings even when prespecified primary endpoints are not met. No prior study in myeloma has systematically examined how primary endpoint results are reported, how closely trial conclusions align with those results, or whether failure to meet a primary endpoint independently predicts the presence of spin after accounting for trial and publication characteristics. We therefore conducted a cross-sectional assessment of RCTs in myeloma in the last 10 years to evaluate the prevalence of spin and its association with primary endpoint reporting and other trial features.

## Methods

### Study design and reporting framework

We conducted a cross-sectional observational analysis of published RCTs in myeloma. Reporting adhered to the Strengthening the Reporting of Observational Studies in Epidemiology (STROBE) guideline for cross-sectional studies.^11^ No patient information was obtained, and data were obtained from publicly available sources; therefore, this study was considered exempt from institutional review board review.

### Protocol registration

The study protocol, including eligibility criteria, outcomes, data abstraction procedures, and the statistical analysis plan, was prospectively registered on the Open Science Framework (OSF) prior to study screening and data extraction (https://osf.io/yube4/metadata/osf). No deviations from the registered protocol occurred.

### Eligibility criteria

Phase I/II, II, or III RCTs evaluating therapeutic interventions for myeloma were included. Eligible trials began enrollment in January 2015 or later, as 2015 marks a turning point in myeloma where the treatment landscape significantly diversified, influencing what treatments were tested. The following types of trials were excluded: nonrandomized studies, single-arm trials, pooled or secondary analyses, trials of smoldering myeloma, and studies focused exclusively on supportive care without randomized therapeutic comparison.

### Search and selection

Trial identification and study selection were based on the prespecified search strategy developed for a prior systematic review of RCTs in myeloma.^2^ Briefly, PubMed/MEDLINE, Embase, and the Cochrane Library were queried for English-language RCTs evaluating therapeutic interventions in patients with myeloma, using a combination of free-text terms related to myeloma and controlled vocabulary (e.g., MeSH terms), with database-specific filters for RCTs. The full electronic search strategies for each database are included in the Supplementary Material.

**Figure 1.**
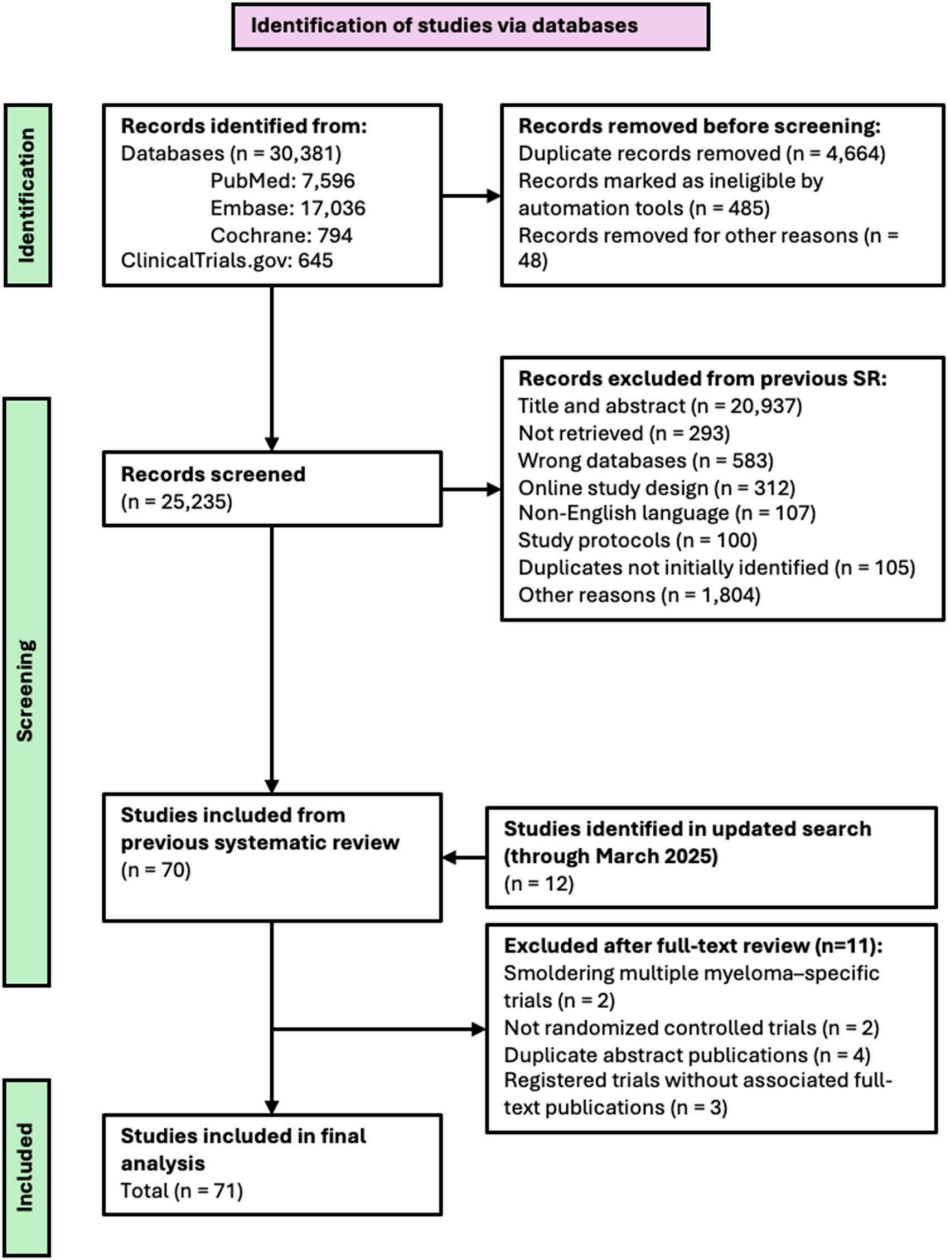
PRISMA flow diagram summarizing the identification, screening, eligibility assessment, and final inclusion of myeloma RCTs. Trials identified through March 2025 underwent the same screening process.

Peer-reviewed, full-text manuscripts, and conference abstracts reporting randomized comparisons of therapeutic interventions for myeloma were included in our study. The initial systematic search was updated through March 2025 for this review. Given that this analysis builds upon a previously conducted and registered systematic review, independent PROSPERO registration was not required. The full original search strategy is publicly available at https://osf.io/mxk8t. Study identification, screening, and inclusion followed the Preferred Reporting Items for Systematic Reviews and Meta-Analyses (PRISMA) 2020 guidelines (Figure 1). Of the 25,235 records screened, 82 trials met the inclusion criteria.  

Two reviewers (M.S. and A.H.S.A.K) independently screened titles and abstracts, followed by full-text review for eligibility. Disagreements were resolved by consensus, with adjudication by a third reviewer when necessary (G.R.M.). When multiple publications referred to the same trial, records were cross-referenced using trial identifiers (ClinicalTrials.gov NCT numbers), and the primary analysis was used.

### Primary endpoint ascertainment

For each included trial, we abstracted the prespecified primary endpoint as stated in the methods section or trial protocol. Primary endpoints were categorized using the same schema as in Mainou et al., according to the conceptual nature of the prespecified primary outcome rather than trial-specific terminology.^2^ Categories included overall survival (OS); progression-based time-to-event endpoints (e.g., PFS, event-free survival); response-based endpoints; minimal residual disease (MRD)–based endpoints; safety or feasibility endpoints; patient-reported outcomes; and other when a single primary outcome could not be clearly identified from the published report. Each reviewer recorded whether the primary endpoint result was statistically significant based on the authors’ prespecified analysis; whether a sample-size calculation was reported in the methods; and whether the primary endpoint and its results were explicitly reported in the abstract and conclusions. Trials terminated early were coded separately when definitive primary endpoint ascertainment was not possible.

### Spin assessment

Spin was defined as reporting practices that distort or overemphasize trial findings in a manner inconsistent with the prespecified primary endpoint.^4^ Using a prespecified coding manual adapted from prior research by Wayant et al., two independent reviewers (M.S. and A.S.) assessed each trial for the presence or absence of spin and classified spin type when present.^5^ Spin categories included secondary endpoint emphasis, subgroup emphasis, optimistic language inconsistent with primary results, unsupported claims of equivalence or clinical benefit, analysis switching, attribution of null results to design limitations, and selective outcome omission. Disagreements were resolved by third-reviewer adjudication (G.R.M.). Operational definitions for spin categories are summarized in Table S1.

### Trial characteristics and journal impact factor

We extracted trial phase, funding source, sample size, medical writer acknowledgement, reporting of OS, year of publication, and enrollment initiation date. Journal impact factor was obtained from the 2024 Clarivate Journal Citation Reports and treated as a continuous variable; no impact factor was coded for abstracts.

### Statistical analysis

Descriptive statistics were used to summarize trial characteristics and the prevalence of spin. Associations between trial characteristics and the presence of spin were evaluated using the chi-square test. Statistical significance was defined as a two-sided *P* < .05. After univariable analyses, 5 variables were selected for multivariable modeling: primary endpoint significance, reporting of a power calculation, trial phase (II vs III), funding structure, and journal impact factor. The journal impact factor was natural-log transformed to account for its right-skewed distribution and reduce the influence of extreme values. These variables were chosen based on univariable associations and prior literature identifying nonsignificant primary outcomes, funding source, and journal characteristics as potential determinants of spin, as well as CONSORT guidance emphasizing transparent sample size reporting.^4^^,^^6^^,^^12^ Given 25 spin events, model inclusion was limited to 5 covariates to preserve an approximate events-per-variable ratio of 5 and reduce overfitting. Trials with nonassessable values were excluded from analyses. Adjusted odds ratios with 95% CIs are reported. Analyses were conducted using R (version 4.5.2).

## Results

### Study identification and inclusion

A total of 82 trials were screened for eligibility. After full-text review, 11 trials were excluded for the following reasons: 2 trials were specific to smoldering myeloma, 2 trials were not RCTs, 4 abstracts represented duplicate publications, and 3 trials were registered without an associated published manuscript or abstract. Following these exclusions, 71 RCTs were included in the final analysis (Figure 1).

### Trial characteristics

Among the included trials, 46 (64.8%) were phase III and 25 (35.2%) were phase II. Thirty-seven trials (52.1%) were funded by pharmaceutical sponsors, and 17 (23.9%) reported mixed (industry and nonprofit) funding. Twelve trials (16.9%) were funded exclusively by nonprofit organizations, and 5 (7.0%) did not report a funding source. Professional medical writing support was acknowledged in 35 trials (49.3%), of which 28 (80.0%) had at least partial pharmaceutical industry sponsorship (Table 1). Nine trials were stopped early (12.7%), and 3 trials (4.2%) employed a noninferiority design. Additional trial details are reported in Table 1.

**Table 1.** Characteristics of included myeloma RCTs (*N* = 71).

Characteristics	No. (%) of trials
**Primary endpoint statistically significant**	
** Yes**	46 (64.8)
** No**	22 (31.0)
** N/A**	3 (4.2)
**Power calculation reported for primary endpoint**	
** Yes**	51 (71.8)
** No**	20 (28.2)
**Primary endpoint discussed in Results section**	
** Yes**	69 (97.2)
** No**	2 (2.8)
**Conclusion aligned with primary endpoint result**	
** Yes**	63 (88.7)
** No**	8 (11.3)
**Overall survival reported**	
** Yes**	50 (70.4)
** No**	21 (29.6)
**Overall survival statistically significant**	
** Yes**	5 (7.0)
** No**	45 (63.4)
** N/A**	21 (29.6)
**Overall survival included in discussion**	
** Yes**	40 (56.3)
** No**	10 (14.1)
** N/A**	21 (29.6)
**Trial stopped early**	
** Yes**	9 (12.7)
** No**	62 (87.3)
**Medical writer involvement**	
** Medical writer acknowledged**	35 (49.3)
** No medical writer acknowledged**	36 (50.7)
**Funding source**	
** Pharmaceutical industry only**	37 (52.1)
** Nonprofit/academic only**	12 (16.9)
** Mixed (industry and nonprofit)**	17 (23.9)
** Other/unclear**	5 (7.1)

### Primary endpoint selection

Progression-based time-to-event endpoints, such as PFS, were the most common primary endpoints, used in 43 of 71 trials (60.6%). Response-based endpoints, including response rate and complete response rate, were designated as the primary endpoint in 19 trials (26.8%), while MRD–based endpoints were used in 6 trials (8.5%). OS was designated as the primary endpoint in one trial (1.4%). Primary endpoints focused on quality of life or patient-reported outcomes were present in one trial (1.4%), as were safety or feasibility-driven primary endpoints (one trial, 1.4%).

### Primary endpoint statistical significance and reporting

The prespecified primary endpoint was statistically significant in 46 trials (64.8%) and not statistically significant in 22 trials (31.0%) (Table 1). In 3 trials (4.2%), the primary endpoint could not be fully assessed due to early study termination. A sample size calculation for the primary endpoint was reported in 51 trials (71.8%).

Nearly all trials (69 of 71; 97.2%) explicitly discussed the primary endpoint in the results section, and in 63 trials (88.7%), the study conclusion aligned with the primary endpoint result. OS was reported as a secondary endpoint in 50 of 71 trials (70.4%) and was statistically significant in 5 (7%). The primary endpoint was statistically significant in 5 of 12 (41.7%) trials with nonprofit funding, 30 of 37 (81.0%) trials with pharmaceutical funding, and 9 of 17 (52.9) trials with mixed funding.

### Prevalence of spin

Of the 71 included trials, 25 (35.2%) exhibited spin (Table 2). The most common manifestation was optimistic language used to characterize unfavorable results, identified in 20 trials (28.2%). This frequently involved describing nonsignificant findings as “encouraging” or noting a “trend toward improvement” despite a lack of statistical significance.^13^^,^^14^ Secondary endpoint emphasis was the second most frequent category, observed in 12 trials (16.9%), in which positive secondary outcomes or exploratory findings were highlighted in the abstract or conclusion to suggest overall benefit despite a null primary endpoint. Among the 25 spin-positive trials, 11 (44.0%) demonstrated more than one spin strategy, most commonly pairing optimistic language with selective emphasis on secondary endpoints or subgroup results.

**Table 2.** Prevalence of spin and spin categories in myeloma RCTs (*n* = 71).

Spin category	Trials with spin, *n* (%)
**Any spin present**	25 (35)
**Optimistic language**	20 (28)
**Secondary endpoint emphasis**	12 (17)
**Subgroup emphasis**	6 (8)
**Unsupported equivalence claims**	5 (7)
**Design-based justification of null results**	5 (7)
**Selective outcome omission**	3 (4)
**Analysis switching**	0 (0)

The examples summarized in Table 3 illustrate patterns of spin in trials with nonsignificant or nonassessable prespecified primary endpoints.^13–17^ Key themes identified were that null or inconclusive primary results were reframed using optimistic language, attributed to design limitations (e.g. early termination or baseline imbalances), or emphasis was instead placed on favorable secondary, subgroup, or exploratory findings. Unsupported suggestions of equivalence or comparability were also observed in the absence of appropriate noninferiority designs. Examples of optimistic language included phases such as “trend favoring,” “encouraging,” or “important option” despite nonsignificant primary outcomes (Table 3).

**Table 3.** Illustrative examples of spin in selected myeloma RCTs.

Study	Primary endpoint result	Spin strategies identified	Illustrative framing
**Dimopoulos et al.^16^**	PFS not significant (HR 0.847; *P* = .477)	Optimistic language; attribution of null results to design limitations	Null result attributed to “immature OS data” and “baseline imbalances”; yet, regimen was described as an “important… option”
**Bashir et al.^17^**	PFS not assessable (early termination)	Optimistic language; attribution of null results to design limitations	Emphasis on “durable disease control” and “well tolerated” despite inability to assess efficacy from early termination
**Huh et al.^15^**	PFS not significant (*P* > .05)	Optimistic language; unsupported equivalence; secondary endpoint emphasis; subgroup emphasis	“Trend favoring Rd,” highlighting tolerability, “durable disease control” and suggesting comparability without noninferiority design
**Voorhees et al.^13^**	PFS not assessable due to redesign mid-study	Secondary endpoint emphasis; optimistic language	Abstract foregrounds overall response rate (51.7%) and describes regimen as “encouraging” despite limited PFS (4.4 months, no reported *P* value)
**Bernal et al.^14^**	Response rate, interim analysis not significant (subgroup *P *= .1)	Subgroup emphasis; optimistic language	Exploratory subgroup described as “trend to higher response”

Representative trials demonstrating common manifestations of spin, including discrepancies between prespecified primary endpoint results and their interpretation in abstracts or conclusions. Spin strategies are categorized according to the study’s prespecified coding framework.

### Association between trial features and spin

**Figure 2.**
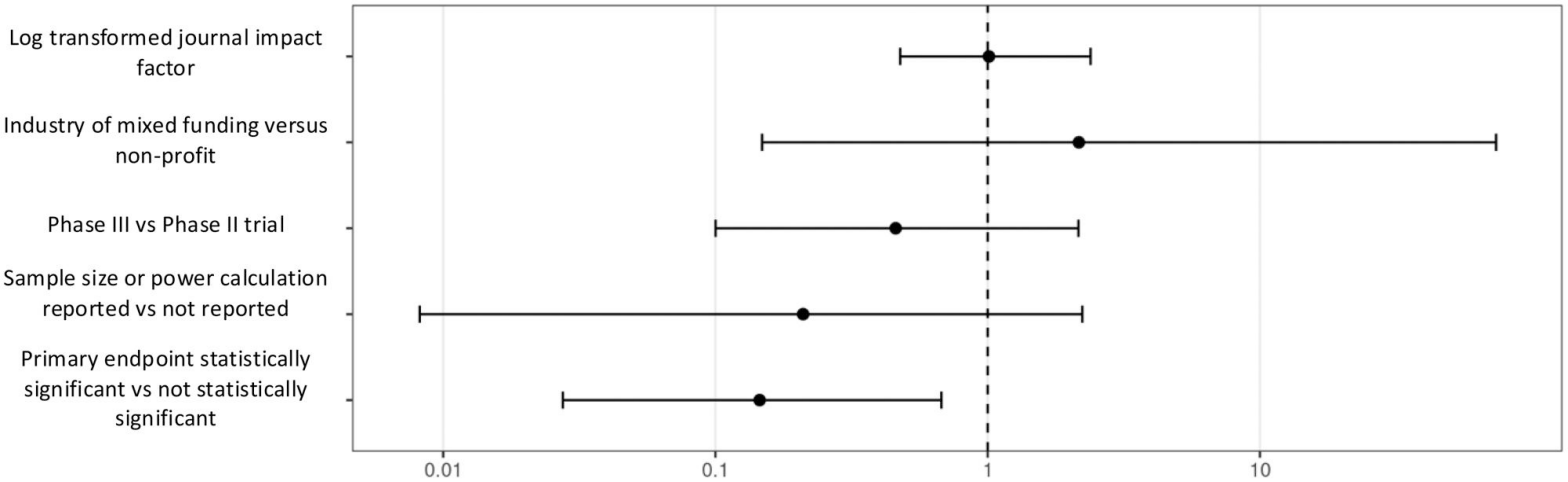
Forest plots depicting outcomes of multivariable analysis of trial characteristics and spin. The *x*-axis represents adjusted odds ratios on a logarithmic scale, with the dashed vertical line indicating no association (OR = 1). Values to the left of 1 indicate lower odds of spin, whereas values to the right of 1 indicate higher odds. Points represent adjusted ORs and horizontal lines denote 95% CIs.

Spin was more frequent among trials with a nonsignificant primary endpoint, absence of a reported power calculation, lower journal impact factor, later publication year, nonprofit funding, phase II design, early stopping, absence of OS reporting or discussion, and lack of an acknowledged medical writer (Table 4). In multivariable analysis controlling for primary endpoint significance, reporting of a power calculation, trial phase, funding structure, and journal impact factor, trials that met their prespecified primary endpoint had significantly lower odds of spin (adjusted OR = 0.15; 95% CI, 0.03-0.70; *P *= .016) (Figure 2). No other variables were statistically significant.

**Table 4.** Association between trial characteristics and presence of spin (*n* = 71).

Trial characteristic	Category	Spin present, *n/N* (%)	*P* value
**Primary endpoint significance**	Nonsignificant	14/22 (64.0)	<.001
	Significant	9/46 (19.6)	
	N/A	2/3 (66.7)	
**Power calculation**	Yes	11/51 (21.6)	<.001
	No	14/20 (70.0)	
**Funding source**	Industry or mixed	14/54 (25.9)	<.001
	Nonprofit	8/12 (66.7)	
	Not reported	3/5 (60)	
**Study phase**	Phase II	10/25 (40.0)	>.01
	Phase III	11/46 (23.9)	
**Overall survival reported**	Yes	16/50 (32.0)	.009
	No	9/21 (42.9)	
**Overall survival included in discussion**	Yes	15/40 (37.5)	.009
	No	1/10 (10.0)	
	N/A	9/21 (42.9)	
**Medical writer acknowledged**	Yes	8/35 (22.9)	.001
	No	17/36 (47.2)	
**Early stopping**	Yes	5/9 (55.6)	.005
	No	20/62 (32.3)	

The N/A category is presented descriptively but was excluded from statistical analyses.

## Discussion

In this cohort study of RCTs in myeloma, we found that spin is common in the interpretation and presentation of trial results. In multivariable analysis, failure to achieve a statistically significant primary endpoint emerged as the sole independent predictor of spin. Given the wide CI, the magnitude of association between primary endpoint significance and spin ranged from very protective to modest. This finding underscores the central role of prespecified primary outcomes and highlights the vulnerability of trial reporting to distortion when primary objectives are not met.

Careful specification and reporting of the prespecified primary endpoint are central to trial interpretability. In this cohort, most myeloma RCTs relied on progression-based time-to-event endpoints, while OS and patient-centered outcomes were infrequently selected as primary measures. Although nearly all trials mentioned the primary endpoint in the results, more than one-quarter (28.2%) did not report a sample size calculation. Sample size calculations specify key design assumptions—effect size, power, and type I error—and allow readers to distinguish true lack of efficacy from underpowered study design.^18^ Prior oncology methodological work has demonstrated that in phase III cancer RCTs, only about one-quarter provided all required parameters for a complete calculation of the primary endpoint sample size.^19^

Our findings align with prior work documenting the prevalence of spin across biomedical research, particularly in randomized trials with nonsignificant primary outcomes. Previous studies have established definitions of spin and demonstrated its frequent presence in abstracts and conclusions of trials with null results,^4^^,^^7^^,^^20^ as well as its capacity to influence clinician interpretation.^21^ However, much of this literature has been descriptive and has pooled trials across heterogeneous disease areas. In oncology, Ito et al. used multivariable logistic regression in a systematic review of noninferiority RCTs to identify trial characteristics independently associated with spin, including treatment novelty and funding source.^10^ Consistent with our findings, Ito et al. reported that spin was present in most non-inferiority oncology trials with nonsignificant primary endpoints (39 of 52 [75%]), a proportion similar to that observed in our cohort (16 of 25 [64%]). They also noted that absence of for-profit funding was associated with higher odds of spin and theorized that the presence of external funding sources would support better trial infrastructure. Similarly, we posit that trials fully reliant on nonprofit funding are more likely to be underpowered and, as a result, negative, which increases the odds of spin and a disproportionate focus on secondary endpoints. Consistent with this, we identified that only 41.7% of trials with exclusively nonprofit funding have statically significant primary endpoints, as opposed to 81.0% of trials with exclusively pharmaceutical funding. Building on this work, our study extends the literature by evaluating a broader cohort of contemporary myeloma RCTs and demonstrating that failure to meet the prespecified primary endpoint is the most important predictor of spin.

Focusing on myeloma provides important context for understanding these dynamics. Myeloma trials frequently rely on surrogate endpoints and are conducted across multiple lines of therapy with heterogeneous patient populations. At the patient level, MRD negativity has been consistently associated with improved PFS and OS, supporting its biological and prognostic relevance.^22^^,^^23^ However, the validity of surrogate endpoints as trial-level predictors of clinically meaningful benefit varies, and improvements in surrogate measures do not consistently translate into gains in OS or quality of life.^24^^,^^25^ Regulatory analyses have shown that oncology therapies approved on the basis of surrogate endpoints often fail to demonstrate durable survival or patient-centered benefit on follow-up.^26^^,^^27^ Consistent with these concerns, we observed lower spin prevalence in trials that reported or discussed OS, supporting arguments for anchoring conclusions to clinically definitive endpoints when available.

Selective emphasis on secondary endpoints and subgroup analyses was a frequent manifestation of spin. In myeloma, secondary outcomes such as OS rate, complete response, very good partial response, and MRD status are routinely assessed and often correlated, but differ in timing, sensitivity, and susceptibility to statistical significance.^28^ Consequently, a single trial may generate multiple favorable signals even when the prespecified primary endpoint is not met, especially if multiple endpoints are analyzed, without adjustments for multiple testing.^21^^,^^29^

Our findings have actionable implications for authors, peer reviewers, and journals. Abstracts and conclusions should explicitly report prespecified primary endpoint results and avoid value-laden language when the primary analysis is statistically nonsignificant, unless clearly labeled as exploratory. When secondary endpoints or subgroup findings are discussed, transparent caveats regarding multiplicity, exploratory intent, and uncertainty are essential. Journal policies and reviewer checklists could further require alignment between primary endpoint results and concluding claims of benefit, consistent with broader initiatives to improve RCT interpretability.^12^

### Limitations

Spin assessment involves judgment; however, we used prespecified operational definitions and independent dual review to mitigate subjectivity. This study also cannot determine intent, and some optimistic language may reflect genuine clinical interpretation rather than intentional misrepresentation. Our analysis was restricted to published trials and conference abstracts; press releases and health media coverage—settings in which spin may be amplified^7^—were not evaluated. Associations should therefore be interpreted as descriptive rather than causal, and residual confounding is possible. Lastly, the limited number of spin events constrained the number of covariates that could be included in multivariable modeling, and the possibility of residual confounding remains. The multivariable analysis should be interpreted cautiously given the limited number of outcome events, with an event-per-variable ratio of 5. Although the adjusted odds ratio for meeting the primary endpoint was statistically significant, the wide CI indicates uncertainty regarding the magnitude of the association.

## Conclusions

In this cross-sectional analysis of RCTs in myeloma, spin was present in approximately one-third of published studies and in nearly two-thirds of trials with nonsignificant primary endpoints. Optimistic language and selective emphasis on secondary or subgroup findings were the most common manifestations. Most contemporary myeloma RCTs relied on progression-based primary endpoints, while OS was inconsistently incorporated into reporting, and a substantial subset failed to document how the primary endpoint sample size was determined—persistent methodological gaps that may increase vulnerability to interpretive spin when primary outcomes are not met. These findings indicate that deviations from primary-endpoint-aligned interpretation remain common in myeloma trial reporting. They highlight the importance of neutral framing and consistency between prespecified primary outcomes and trial conclusions to support clear and reliable interpretation of trial results.

## Supplementary Material

oyag221_Supplementary_Data

## Data Availability

Data can be shared upon reasonable request to the corresponding author.
