# Supplementary material for: Reporting of primary endpoint and associated spin in randomized myeloma trials
Source: Oncologist. 2026 Jun 2;31(7):oyag221. doi: 10.1093/oncolo/oyag221 (PMC13275311; doi:10.1093/oncolo/oyag221)
Supplement: oyag221_Supplementary_Data [file oyag221_supplementary_data.docx]

## **Search Strategy**

## PUBMED:

(((("Multiple Myeloma"[Mesh]) OR "Plasmacytoma"[Mesh]) OR (multiple myeloma OR plasmacytoma OR plasmacytom* OR myelom*))) AND ((randomized controlled trial[pt] OR controlled clinical trial[pt] OR randomized[tiab] OR placebo[tiab] OR clinical trials as topic[mesh:noexp] OR randomly[tiab] OR trial[ti] NOT (animals[mh] NOT humans [mh])))

## COCHRANE: (limited to trials)

MeSH descriptor: [Multiple Myeloma] explode all trees MeSH descriptor: [Plasma Cells] explode all trees

Multiple myeloma:ti,ab,kw Plasmatocytoma:ti,ab,kw Plasmatocytom*:ti,ab,kw Myelom*:ti,ab,kw

## EMBASE:

Ab(Multiple Myeloma[Mesh]) OR ab(“multiple myeloma”) OR ab(plasmacytoma*) OR ab(myelom*) OR ab(Plasmacytoma [Mesh])

AND (su.exact.explode(“clinical trial” OR “clinical trial (topic)” OR “clinical trials as topic”) OR qu(“clinical trial”) OR dtype,ti,su,subst(“clinical trial” OR “clinical trials” OR “clin trial” OR “equivalence trial” OR “equivalence trials” OR “multicenter study” OR “multicenter studies” OR “randomized controlled trial” OR “randomized controlled trials”))

AND ((human OR humans OR man OR men OR women OR woman OR patient OR patients OR volunteer OR volunteers OR “homo sapiens” OR Hominidae OR male OR males OR female OR females OR adult OR adults))

## **Supplemental Table 1. Operational Definitions of Spin in Myeloma Randomized Clinical Trials**

| **Spin Category** | **Operational Definition** |
| --- | --- |
| **Secondary endpoint emphasis** | Preferential emphasis on statistically significant secondary or surrogate endpoints (e.g., response rate, depth of response, minimal residual disease) when the prespecified primary endpoint was not statistically significant. |
| **Subgroup emphasis** | Emphasis on statistically significant subgroup, post-hoc, or modified-population analyses to imply overall trial efficacy when the overall trial result was negative. |
| **Equivalence claims without statistical support** | Interpretation of a nonsignificant P value in a superiority trial as evidence of noninferiority or equivalence without a prespecified and met noninferiority or equivalence design. |
| **Analysis switch** | Emphasis on non-prespecified analyses, such as per-protocol or exploratory analyses, when intention-to-treat analysis was prespecified. |
| **Optimistic language** | Use of assertive or suggestive language that overstates benefit when the overall trial is negative (e.g., “clinically meaningful,” “trend toward benefit,” “new standard of care,” “effective and well tolerated”). |
| **Attribution of null results to design limitations** | Attribution of nonsignificant primary results to trial design limitations (e.g., underpowering, crossover, short follow-up) while still implying treatment benefit. |
| **Selective omission** | Omission or downplaying of nonsignificant or unfavorable patient-important outcomes, particularly overall survival, in abstracts or conclusions. |
